# Editorial: Pulmonary hypertension: from bench to bedside

**DOI:** 10.3389/fphys.2024.1421654

**Published:** 2024-05-08

**Authors:** Vasile Foris, Andrea Olschewski

**Affiliations:** ^1^ Division of Pulmonology, Department of Internal Medicine, Medical University of Graz, Graz, Austria; ^2^ Ludwig Boltzmann Institute for Lung Vascular Research, Graz, Austria; ^3^ Experimental Anesthesiology, Department of Anesthesiology and Intensive Care Medicine, Medical University of Graz, Graz, Austria

**Keywords:** pulmonary hypertension, biomarker, multiomics, metabolomics, ARDS

An exciting challenge in the field of pulmonary hypertension (PH) is the translation of *in vitro, in silico* and *in vivo* findings from the bench to the bedside. Given the complexity and the burden of the disease, we have sought contributions in this Research Topic that address this challenge. The importance of this Research Topic relies on its multidisciplinary views on the physiology and pathophysiology of the pulmonary vasculature including both original papers and reviews.

Since the recent European Society of Cardiology/European Respiratory Society guidelines for the diagnosis and treatment of pulmonary hypertension have been published, there is increasing evidence about the importance of exercise induced hemodynamic changes (Humbert et al., 2022; Baratto et al., 2023; Karvasarski et al., 2023; Sherman and Saggar, 2023). It is now recognized that pulmonary vascular reserve may not be as accurately described by right heart catheterization (RHC) at rest as it would be with RHC during exercise (Forbes et al., 2023; Tello et al., 2023). Additionally, there is a growing body of data on the metabolic changes that are triggered or accompanied by exercise in PH (Simpson et al., 2023). The possible interactions between pulmonary vascular metabolism and exercise physiology in the context of PH have been reviewed by Lee et al. Furthermore, they describe an international collaborative project aimed at deciphering exercise-induced metabolic changes in PH. They propose an elegant protocol in which blood is drawn from both the pulmonary artery and the radial artery in patients with PH undergoing clinically indicated RHC. Applying metabolomics methods, they are going to interrogate the potential of transpulmonary metabolomics to better understand the role and impact of exercise induced changes in the pulmonary vasculature. Since most patients affected by PH ultimately die of right heart failure it is crucial to understand the underlying mechanisms of the failing heart. Hindmarch et al. sought to leverage the metabolic pattern of the failing heart in a rat model of monocrotaline induced PH using a multiomics approach (Hindmarch et al.). By integrating previously published RNA sequencing data with the newly identified proteins from the harvested cardiac tissue, several transcript-protein pairs that may contribute to right heart failure were identified. Functional annotation of these pairs revealed the substantial involvement of purine metabolism, mitochondrial function, and cellular respiration. These observations were validated on independent publicly available datasets originating from other investigators. In addition, the authors describe in detail fifteen highly enriched proteins that also have highly enriched transcripts adding weight to previous studies linking these targets to PH. An interesting approach would be to test the circulating level of these proteins in PH patients to answer the question of whether they could serve as biomarkers of adaptive or maladaptive right ventricular failure. Many efforts have been undertaken to identify suitable blood derived biomarkers that aid diagnosis and/or risk stratification and/or treatment decisions (Foris et al., 2013). Despite many candidates only brain natriuretic peptide (BNP) and N-Terminal pro-B-Type Natriuretic peptide (NT-proBNP) have both scalable applications on standard clinical laboratory platforms and a role in risk stratification scores for PAH. Aiming to discover and validate additional biomarkers Foris et al. assessed apelin isoforms in a series of carefully phenotyped prospectively collected patients with idiopathic pulmonary arterial hypertension (IPAH), chronic thromboembolic pulmonary hypertension (CTEPH) and healthy controls (Foris et al.). The key finding of the study was that apelin-17 was elevated in the sera of both the IPAH and CTEPH cohorts and it was the most abundant isoform. A model was developed that includes apelin −17, NT-proBNP and growth differentiation factor 15 (GDF-15) that can detect IPAH pointing towards the potential role of apelin-17 as a diagnostic biomarker. The role of the apelinergic system in the pulmonary circulation has been a Research Topic of intensive research. Apelin has been shown to suppress proliferation and induced apoptosis of pulmonary arterial smooth muscle cells. Moreover, apelin has been identified as a transcriptional target that is downregulated in pulmonary endothelial cells from PAH patients with deficient bone morphogenetic protein receptor type 2 (BMPR2) expression (Alastalo et al., 2011). Therefore, apelin is a desirable drug target. Interfering with the apelin-BMPR2 pathway could have a direct translational impact, as germline mutations of the *BMPR2* gene are responsible for approximately 80% of hereditary PH cases (Eichstaedt et al., 2023).

Pulmonary endothelial dysfunction can occur not only due to mutations or dysfunctions of the *BMPR2* gene. In fact, there are currently at least 27 genes with putative evidence of PAH pathogenicity with varying degrees of penetrance (Welch et al., 2023). Additional factors that contribute to endothelial dysfunction, such as shear stress, further worsen the loss of pulmonary endothelial tight junctions. Garcia-Flores et al. investigated the impact of mechanical stress on SRY-Box Transcription Factor 18 (SOX18) which is important for the regulation of vascular permeability and claudin 5, an endothelial tight junction protein in human lung microvascular endothelial cells (Garcia-Flores et al.). They found that SOX18 and Claudin-5 are downregulated in human lung microvascular endothelial cells exposed to cyclic stretch. This is further evidence of their previous findings where they showed that SOX18 expression is increased by shear stress in lung endothelium (Gross et al., 2014). Targeted overexpression of SOX18 in mice ventilated with high tidal volumes resulted in preserved claudin-5 expression and in reduced pulmonary vascular leakage. Overall, these results suggest that enhancing SOX18 expression may be a potential approach to treat ventilator-induced lung injury (VILI), however there are potential avenues for future research. Felix et al. used a rat model of acute respiratory distress syndrome (ARDS) to study the impact of different fluid strategies and low or high positive end-expiratory pressure (PEEP) on VILI (Felix et al.). The authors found that a more liberal fluid strategy and high PEEP caused greater perivascular edema and that the changes were detrimental not only to the lungs but also to the kidneys. The findings are based on detailed hemodynamic measurements as well as histologic and gene expression analyses.

We believe that the compilation of these contributions highlights the value of interdisciplinary collaboration and the power of collective knowledge in the dynamic field of PH. We hope that these findings will collectively advance the domain and help the community foster knowledge sharing and that this Research Topic may inspire other investigators in this scientific area.
